# One complete and three draft genome sequences of four *Brochothrix thermosphacta* strains, CD 337, TAP 175, BSAS1 3 and EBP 3070

**DOI:** 10.1186/s40793-018-0333-z

**Published:** 2018-10-10

**Authors:** Nassima Illikoud, Christophe Klopp, Alain Roulet, Olivier Bouchez, Nathalie Marsaud, Emmanuel Jaffrès, Monique Zagorec

**Affiliations:** 1 UMR 1014 SECALIM, INRA, Oniris, Nantes, France; 2 Plateforme Bio-informatique, Toulouse Genopole, Institut National de la Recherche Agronomique, Castanet-Tolosan, France; 3 INRA, US 1426, GeT-PlaGe, Genotoul, Castanet-Tolosan, France; 4 LISBP, Université de Toulouse, CNRS, INRA, INSA, Toulouse, France

**Keywords:** *Brochothrix thermosphacta*, *Listeriaceae*, Spoilage, Chicken meat, Cooked shrimp, Bovine slaughterhouse, smoked salmon

## Abstract

*Brochothrix thermosphacta* is one of the dominant bacterial species associated with spoilage of chilled meat and seafood products through the production of various metabolites responsible for off-odors. However, metabolic pathways leading to meat and seafood spoilage are not all well known. The production of spoiling molecules seems to depend both on strains and on food matrix. Several *B. thermosphacta* genome sequences have been reported, all issued from meat isolates. Here, we report four genome sequences, one complete and three as drafts. The four *B. thermosphacta* strains CD 337, TAP 175, BSAS1 3, and EBP 3070 were isolated from different ecological niches (seafood or meat products either spoiled or not and bovine slaughterhouse). These strains known as phenotypically and genetically different were selected to represent intraspecies diversity. CD 337 genome is 2,594,337 bp long, complete and circular, containing 2593 protein coding sequences and 28 RNA genes. TAP 175, BSAS1 3, and EBP 3070 genomes are arranged in 57, 83, and 71 contigs, containing 2515, 2668, and 2611 protein-coding sequences, respectively. These genomes were compared with two other *B. thermosphacta* complete genome sequences. The main genome content differences between strains are phages, plasmids, restriction/modification systems, and cell surface functions, suggesting a similar metabolic potential but a different niche adaptation capacity.

**Electronic supplementary material:**

The online version of this article (10.1186/s40793-018-0333-z) contains supplementary material, which is available to authorized users.

## Introduction

*Brochothrix* and *Listeria* are the only two genera belonging to the *Listeriaceae* family. *Brochothrix thermosphacta* is a non-pathogenic, psychrotrophic, and ubiquitous bacterial species. It is responsible for the spoilage of chilled meat and seafood products stored aerobically or under modified atmosphere or vacuum packaging. Spoilage of these foodstuffs by *B. thermosphacta* results from the production of volatile organic compounds (VOCs) responsible for off-odors. Two VOCs, 3-hydroxy-2-butanone (acetoin) and 2,3-butanedione (diacetyl) have been associated with both meat and seafood products spoilage, whatever the packaging conditions [1]. We recently showed that the concentration of acetoin and diacetyl produced in beef or shrimp juices was strain and matrix dependent although the differences in the production level did not depend on the ecological origin of the strains [2]. The presence of other VOCs associated to the presence of *B. thermosphacta* in food depends on the food product (meat or seafood) and on the storage conditions [1]. Thus, it is yet unknown whether strains isolated from meat or from seafood have a similar spoilage potential and/or whether the food matrix or storage conditions can impact the expression of this potential. To date, 14 *B. thermosphacta* genome sequences, including two complete, are publicly available [3, 4]. A comparative genomic analysis on 12 *B. thermosphacta* draft genome sequences showed a high degree of similarity between strains and a similar gene repertoire for the production of several malodorous molecules [3]. Major gene repertoire differences among the genomes previously reported in the literature were characterized by phage related sequences [3, 4]. However, all were isolated from meat products and thus may not represent exhaustive *B. thermosphacta* diversity since this species has also been isolated from other ecological niches [5]. In addition, although *B. thermosphacta* has been reported to be involved in a wide variety of foodstuff spoilage, metabolic pathways responsible for most of the produced VOCs still remain unknown. In this study we chose four *B. thermosphacta* strains issued from diverse environments for a comparative genomic analysis. *B. thermosphacta* CD 337, TAP 175, EBP 3070, and BSAS1 3 were isolated from spoiled cooked and peeled shrimp, fresh chicken leg, spoiled smoked salmon, and from a bovine slaughterhouse environment (hide of a slaughtered animal), respectively [2]. These strains were chosen to represent a larger diversity than previously explored in genome comparisons. Indeed we previously showed that the four chosen strains are genotypically distant and have different abilities to produce acetoin and diacetyl [2]. Gene content of these four strains was compared to those of 14 available *B. thermosphacta* genome sequences in order to screen for potential features associated to specific niche adaptation or fitness, and for putative differences in their spoilage potential.

## Organism information

### Classification and features

**Fig. 1:**
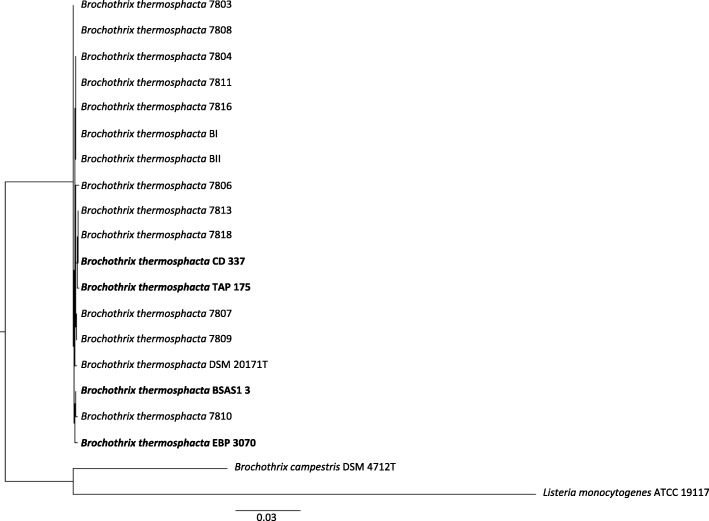
Phylogenetic tree showing the relationship of the four *Brochothrix thermosphacta* strains (shown in bold print) to other *B. thermosphacta* strains the genome of which is publically available. *Brochothrix campestris*, and *Listeria monocytogenes* type strains were used as outgroup. Tree is based on MAFFT (v7.309) [42] aligned complete *rpoB* gene sequences. The tree was built using FastTree (v2.1.5) then visualized with FigTree (v1.4.3)

*B. thermosphacta* CD 337, TAP 175, EBP 3070, and BSAS1 3 were isolated after plating on *B. thermosphacta* selective medium Streptomycin-thallous acetate-actidione (STAA, Oxoid) [6, 7]. Strains belonging to *B. thermosphacta* species were described as Gram positive, non-spore forming, and non-motile regular unbranched rods (Table 1). They are aerobe and facultative anaerobe, catalase positive and oxidase negative [5]. Gram staining and catalase reaction of freshly-grown cells of *B. thermosphacta* CD 337, TAP 175, EBP 3070, and BSAS1 3 confirmed all to be Gram positive and catalase positive [2]. Phylogenetic analyses based on *rpoB* gene sequence alignments (Fig. 1) showed that *B. thermosphacta* strains CD 337, TAP 175, EBP 3070 and BSAS1 3 clustered within the *Brochothrix* genus. Based on these analyses, our four *B. thermosphacta* were also found to be closely related to *Brochothrix campestris* the only other species yet described in the *Brochothrix* genus and to *Listeria monocytogenes*.

**Table 1:** Classification and general features of B. thermosphacta strains CD 337, TAP 175, BSAS1 13, and EBP 3070

MIGS ID	Property	CD 337		TAP 175		BSAS1 3		EBP 3070	
		Term	Evidence code^a^	Term	Evidence code^a^	Term	Evidence code^a^	Term	Evidence code^a^
	Classification	Domain *Bacteria*	TAS [32]	Domain *Bacteria*	TAS [32]	Domain *Bacteria*	TAS [32]	Domain *Bacteria*	TAS [32]
		Phylum *Firmicutes*	TAS [33, 34]	Phylum *Firmicutes*	TAS [33, 34]	Phylum *Firmicutes*	TAS [33, 34]	Phylum *Firmicutes*	TAS [33, 34]
		Class *Bacilli*	TAS [35]	Class *Bacilli*	TAS [35]	Class *Bacilli*	TAS [35]	Class *Bacilli*	TAS [35]
		Order *Bacillales*	TAS [36]	Order *Bacillales*	TAS [36]	Order *Bacillales*	TAS [36]	Order *Bacillales*	TAS [36]
		Family *Listeriaceae*	TAS [37]	Family *Listeriaceae*	TAS [37]	Family *Listeriaceae*	TAS [37]	Family *Listeriaceae*	TAS [37]
		Genus *Brochothrix*	TAS [38]	Genus *Brochothrix*	TAS [38]	Genus *Brochothrix*	TAS [38]	Genus *Brochothrix*	TAS [38]
		Species *Brochothrix thermosphacta*	TAS [38]	Species *Brochothrix thermosphacta*	TAS [38]	Species *Brochothrix thermosphacta*	TAS [38]	Species *Brochothrix thermosphacta*	TAS [38]
		Strain CD 337	TAS [2]	Strain TAP 175	TAS [2]	Strain BSAS1 3	TAS [2]	Strain EBP 3070	TAS [2]
	Gram stain	Positive	IDA	Positive	IDA	Positive	IDA	Positive	IDA
	Cell shape	Rod	IDA	Rod	IDA	Rod	IDA	Rod	IDA
	Motility	Non-motile	NAS [5]	Non-motile	NAS [5]	Non-motile	NAS [5]	Non-motile	NAS [5]
	Sporulation	Non-sporulating	NAS [5]	Non-sporulating	NAS [5]	Non-sporulating	NAS [5]	Non-sporulating	NAS [5]
	Temperature range	0–30 °C	NAS [5]	0–30 °C	NAS [5]	0–30 °C	NAS [5]	0–30 °C	NAS [5]
	Optimum temperature	20–25 °C	NAS [5]	20–25 °C	NAS [5]	20–25 °C	NAS [5]	20–25 °C	NAS [5]
	pH range; Optimum	5–9; 7	NAS [5]	5–9; 7	NAS [5]	5–9; 7	NAS [5]	5–9; 7	NAS [5]
	Carbon source	Glucose, ribose, glycerol, mannose, mannitol, gluconate, glucosamine, fructose, maltose, sucrose, trehalose	NAS [39]	Glucose, ribose, glycerol, mannose, mannitol, gluconate, glucosamine, fructose, maltose, sucrose, trehalose	NAS [39]	Glucose, ribose, glycerol, mannose, mannitol, gluconate, glucosamine, fructose, maltose, sucrose, trehalose	NAS [39]	Glucose, ribose, glycerol, mannose, mannitol, gluconate, glucosamine, fructose, maltose, sucrose, trehalose	NAS [39]
MIGS-6	Habitat	Cooked and peeled spoiled shrimp	TAS [2]	Non-spoiled chicken leg	TAS [2]	Beef slaughterhouse environment	TAS [2]	Spoiled smoked salmon	TAS [2]
MIGS-6.3	Salinity	Tolerate 8–10% NaCl (*w*/*v*)	NAS [5]	Tolerate 8–10% NaCl (w/v)	NAS [5]	Tolerate 8–10% NaCl (w/v)	NAS [5]	Tolerate 8–10% NaCl (w/v)	NAS [5]
MIGS-22	Oxygen requirement	Facultative anaerobe	NAS [5]	Facultative anaerobe	NAS [5]	Facultative anaerobe	NAS [5]	Facultative anaerobe	NAS [5]
MIGS-15	Biotic relationship	free-living	NAS	free-living	NAS	free-living	NAS	free-living	NAS
MIGS-14	Pathogenicity	Non-pathogenic	NAS [5]	Non-pathogenic	NAS [5]	Non-pathogenic	NAS [5]	Non-pathogenic	NAS [5]
MIGS-4	Geographic location	Pays de Loire, France	NAS	Pays de Loire, France	NAS	Pays de Loire, France	NAS	Pays de Loire, France	NAS
MIGS-5	Sample collection	2009	TAS [40]	February, 2014	TAS [2]	April, 2015	TAS [2]	June, 2011	NAS [2]
MIGS-4.1	Latitude	47.2173° N	NAS	47.2173° N	NAS	47.059° N	NAS	47.2173° N	NAS
MIGS-4.2	Longitude	1.5534° W	NAS	1.5534° W	NAS	0.876° W	NAS	1.5534° W	NAS
MIGS-4.4	Altitude	2–52; 20 m	NAS	2–52; 20 m	NAS	63–184; 100 m	NAS	2–52; 20 m	NAS

^a^Evidence codes - IDA: Inferred from Direct Assay; TAS: Traceable Author Statement (i.e., a direct report exists in the literature); NAS: Non-traceable Author Statement (i.e., not directly observed for the living, isolated sample, but based on a generally accepted property for the species, or anecdotal evidence). These evidence codes are from the Gene Ontology project [41]. When the evidence is IDA, the property was directly observed for a live isolate

**Fig. 2:**
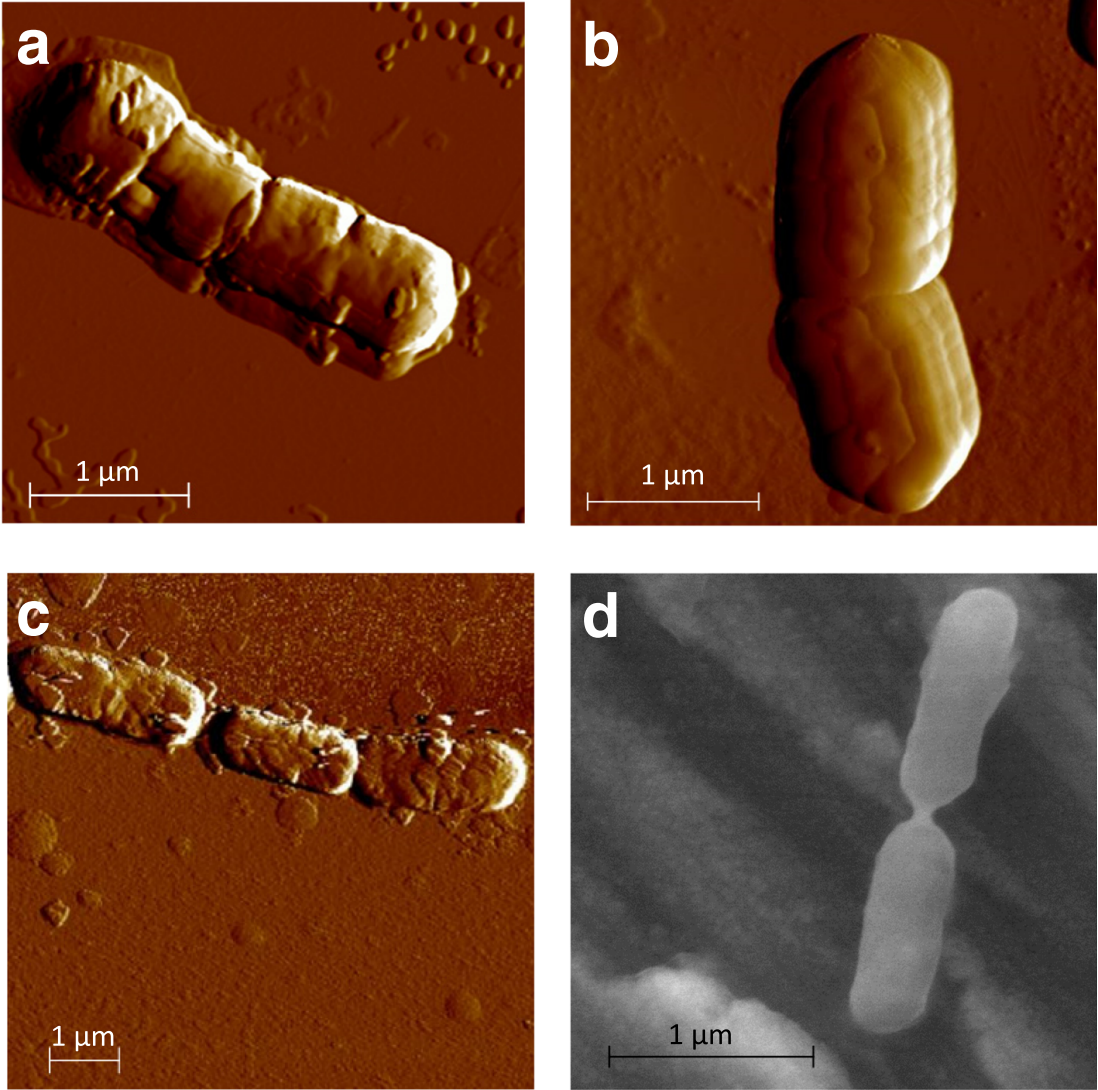
Microscopy pictures of liquid-grown *Brochothrix thermosphacta* cultures. *Brochothrix thermosphacta* CD 337 (**a**), *B. thermosphacta* EBP 3070 (**b**), *B. thermosphacta* BSAS1 3 (**c**) were imaged by atomic force microscopy, and *B. thermosphacta* TAP 175 (**d**) was generated by scanning electron microscopy. The scale bars represent 1 μm

Atomic force and scanning electron microscopies of fresh cultures showed that each strain population consisted mainly cells that were rod shaped with no flagella (Fig. 2).

#### Extended feature descriptions

The four strains were previously genetically and phenotypically characterized. These strains isolated from different ecological niches, belonged to different Rep-PCR, PFGE and MALDI-TOF clusters and present different abilities to produce diacetyl and acetoin in beef and cooked shrimp juices [2]. On the whole strain collection tested, CD 337 and BSAS1 3 were within the highest acetoin and diacetyl producers when grown in beef juice, and among the lowest after growth in cooked shrimp juice. Conversely TAP 175 and EBP 3070 produced the highest levels of acetoin and diacetyl in shrimp juice and the lowest ones after cultivation in beef juice [2].

## Genome sequencing information

### Genome project history

In order to investigate *B. thermosphacta* species diversity, an initial study has been conducted on 159 isolates issued from various ecological niches. They were phenotyped on their ability to produce acetoin and diacetyl and were clustered, based on their genotypes (PFGE, Rep-PCR) and proteomic (MALDI-TOF) patterns [2]. Strains did not cluster based on their ecological origin nor on their spoilage compounds production ability. Therefore, to determine whether the spoilage potential of the strains was due to their gene repertoire and/or to the food matrix, we selected 4 strains, as diverse as possible, for a comparative genomic analysis. *B. thermosphacta* CD 337, TAP 175, BSAS1 3, and EBP 3070 have been isolated from a variety of food products and from environment. They have different abilities to produce spoiling molecules depending on the food matrix, and belong to different PFGE and Rep-PCR clusters [2]. Project information and associated MIGS are shown in Table 2.

**Table 2:** Project information

MIGS ID	Property	Term, CD 337	Term, TAP 175	Term, BSAS1 3	Term, EBP 3070
MIGS 31	Finishing quality	Complete circular genome	Draft genome	Draft genome	Draft genome
MIGS-28	Libraries used	20-kb Template Preparation Using BluePippin Size-Selection System (15-kb size cutoff)	NEB Next Fast DNA fragmentation and library prep (NEB Biolabs)	NEB Next Fast DNA fragmentation and library prep (NEB Biolabs)	NEB Next Fast DNA fragmentation and library prep (NEB Biolabs)
MIGS 29	Sequencing platforms	PacBio RSII (Pacific BioSciences)	Ion S5 (Ion Torrent)	Ion S5 (Ion Torrent)	Ion S5 (Ion Torrent)
MIGS 31.2	Fold coverage	526.48	270.67	206.01	243.65
MIGS 30	Assemblers	CANU version 1.3	SPAdes (version 3.9.0)	SPAdes (version 3.9.0)	SPAdes (version 3.9.0)
MIGS 32	Gene calling method	MicroScope Genoscope Plateform [12]	MicroScope Genoscope Plateform [12]	MicroScope Genoscope Plateform [12]	MicroScope Genoscope Plateform [12]
	Locus Tag	BTCD	BTTAP	BTBSAS	BTEBP
	Genbank ID	ERZ500814	ERZ500815	ERZ500816	ERZ500817
	GenBank Date of Release	25 May 2018	25 May 2018	25 May 2018	25 May 2018
	GOLD ID				
	BIOPROJECT	PRJEB25018	PRJEB25018	PRJEB25018	PRJEB25018
MIGS 13	Source Material Identifier				
	Project relevance	Food spoiler	Food spoiler	Food spoiler	Food spoiler

### Growth conditions and genomic DNA preparation

*B. thermosphacta* strains were grown overnight at 25 °C in l00 ml Luria-Bertani Broth (Invitrogen) containing 10 g/l NaCl. Cultures were shaken at 100 rpm. A cell pellet (about 2 × 10^10^ CFU) was obtained by centrifugation at 5000 x *g* for 10 min. Genomic DNA was extracted using the Blood and Cell Culture DNA Midi Kit (Qiagen, France) according to the manufacturer’s instructions for Gram positive bacteria with some modifications as previously described by [8]. Briefly, the cell pellet was resuspended in 3.5 ml buffer B1 containing 0.2 mg/ml RNAse A. Bacterial cells were lysed by the addition of 220 mg lysozyme powder (Euromedex, France) followed by incubation for 2 h at 37 °C. High molecular weight genomic DNA was purified by gravity flow and anion exchange chromatography, eluted in 5 ml QF buffer (Qiagen) and precipitated with 3.5 ml isopropanol. DNA was collected by centrifugation for 15 min at 4 °C and 10,000 x *g* and then air dried for 10 min. DNA was resuspended in 100 μl TE buffer (10 Mm Tris-HCl, 1 mM EDTA, pH 8.0) for two hours at 55 °C. DNA integrity was checked on a 0.8% agarose gel. DNA concentration and purity were checked using Nanodrop spectrophotometer 2000 (Thermo Scientific). The ratio 260 nm and 280 nm was assessed to be 1.9.

### Genome sequencing and assembly

*B. thermosphacta* CD 337 sequence reads were generated at GeT-PlaGe (Plateforme Génomique), INRA Auzeville, France with a single-molecule-real-time (SMRT) using Pacific Biosciences RS II sequencing technology (Table 2). A total of 113,824 reads was produced. *De-novo* assembly was carried out using CANU version 1.3 with standard parameters [9]. Raw data were aligned then polished with pbalign and quiver de smrtshell-2.3.0, respectively. The resulting contig was circularized with circlator (version 1.3.0) [10]. For the three other strains TAP 175, BSAS1 3, and EBP 3070, library preparation and genome sequencing were carried out at GeT-Biopuces platform (INSA, Toulouse, France) using S5 sequencer from Ion Torrent technology (Table 2). The resulted reads, approximately 2.60, 2.26, and 2.88 million for TAP 175, BSAS1 3 and EBP 3070, respectively, were *de-novo* assembled using SPAdes (version 3.9.0) with default parameters [11]. The assembly resulted in 57, 83, and 71 contigs, respectively.

### Genome annotation

The new complete and draft genome sequences were integrated in the MicroScope platform hosted in the Genoscope for automatic annotation [12]. This tool uses multiple databases: TrEMBL, SwissProt, FigFam, PubMed, InterPro, etc. The Microscope platform also provides links to databases as PkGDB, MicroCyc, KEGG for extracting genomic and metabolic data from the pathway genome database [12]. Expert annotation was performed for all the genes of *B. thermosphacta* CD 337 genome using the gene annotation editor. Expert manual annotations were then transferred from CD 337 on close orthologs (i.e. > 90% identity on > 80% length or > 85% identity when syntheny was observed) of the draft genomes from the three other *B. thermospacta* strains.

## Genome properties

**Fig. 3:**
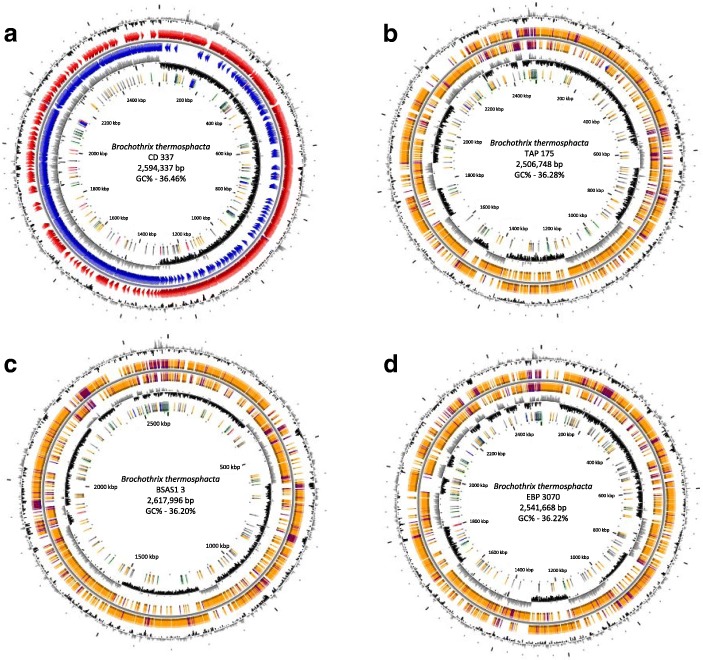
Circular views of genome sequences of *Brochothrix thermosphacta* CD 337 (**a**), *B. thermosphacta* TAP 175 (**b**), *B. thermosphacta* BSAS1 3 (**c**), and *B. thermosphacta* EBP 3070 (**d**). The circular display shows, from outside to inside: (i) GC percentage; (ii) Predicted CDSs transcribed in the clockwise direction; (iii) Predicted CDSs transcribed in the counterclockwise direction. In (ii) and (iii), red and blue colors represent MaGe validated annotations, orange color represents the MicroScope automatic annotation with a reference genome, and the purple color represents Primary/Automatic annotations; (iv) GC skew (G + C/G-C) and (v) color-code representing rRNA (blue), tRNA (green), miscellaneous RNA (orange), Transposable elements (pink) and pseudogenes (grey)

The circular genome of *B. thermosphacta* CD 337 is 2,594,337 nucleotides with a 36.46% GC content (Table 3) and contains one finalized chromosome (Fig. 3). This genome contains 2943 protein coding sequences (CDS). The draft genomes of strains TAP 175, BSAS1 3, and EBP 3070 consist in 57, 83, and 71 contigs, respectively. The genome of *B. thermosphacta* TAP 175 has an estimated size of 2,506,748 bp, with a 36.28% GC content. That of *B. thermosphacta* BSAS1 3 encompasses 2,617,996 bp (36.20% G + C), and that of the strain EBP 3070 is 2,541,668 bp long (36.22% G + C) (Fig. 3). The three genomes contain 2515, 2668, and 2611 CDS, respectively. The genome properties and statistics are summarized in Table 3, and the number of genes assigned to COG functional categories in Table 4.

**Table 3:** Genome statistics

Attribute	CD 337		TAP 175		BSAS1 3		EBP 3070	
	Value	% of Total	Value	% of Total	Value	% of Total	Value	% of Total
Genome size (bp)	2,594,337	100	2,506,748	100	2,617,996	100	2,541,668	100
DNA coding (bp)	2,182,130	84.11	2,136,251	85.22	2,213,516	84.55	2,118,751	83.36
DNA G + C (bp)	945,895	36.46	909,448	36.28	947,714	36.20	920,592	36.22
DNA scaffolds	1	100	57	100	83	100	71	100
Total genes	2743	100	2638	100	2796	100	2740	100
Protein coding genes	2593	94.53	2515	95.3	2668	95.4	2611	95.3
RNA genes	28	1.0	6	0.2	8	0.3	9	0.3
Pseudo genes	38	1.4	43	1.6	57	2.0	102	3.7
Genes in internal clusters	N/D	–	N/D	–	N/D	–	N/D	–
Genes with function prediction	2031	74.04	1928	73.1	1957	70.0	1954	71.3
Genes assigned to COGs	2486	90.6	2429	92.1	2472	88.4	2432	88.7
Genes with Pfam domains	2026	73.9	1982	75.1	2040	73.0	2002	73.1
Genes with signal peptides	85	3.1	83	3.1	83	3.0	85	3.1
Genes with transmembrane helices	392	14.3	384	14.5	386	13.8	389	14.2
CRISPR repeats	1	0.04	2	0.07	1	0.03	0	0

**Table 4:** Number of genes associated with general COG functional categories

Code	CD 337		TAP 175		BSAS1 3		EBP 3070		Description
	Value	%age	Value	%age	Value	%age	Value	%age	
J	158	6.09	161	6.40	159	5.96	164	6.28	Translation, ribosomal structure and biogenesis
A	ND	–	ND	–	ND	–	ND	–	RNA processing and modification
K	237	9.14	235	9.34	242	9.07	234	8.96	Transcription
L	131	5.05	125	4.97	139	5.21	130	4.98	Replication, recombination and repair
B	ND	–	ND	–	ND	–	ND	–	Chromatin structure and dynamics
D	35	1.35	35	1.39	36	1.35	34	1.30	Cell cycle control, Cell division, chromosome partitioning
V	63	2.43	55	2.19	52	1.95	57	2.18	Defense mechanisms
T	100	3.86	95	3.74	98	3.67	99	3.79	Signal transduction mechanisms
M	117	4.51	106	4.21	119	4.46	109	4.17	Cell wall/membrane biogenesis
N	16	0.62	12	0.48	17	0.64	15	0.57	Cell motility
U	33	1.27	27	1.07	29	1.09	28	1.07	Intracellular trafficking and secretion
O	71	2.74	69	2.74	66	2.47	69	2.64	Posttranslational modification, protein turnover, chaperones
C	106	4.09	106	4.21	107	4.01	105	4.02	Energy production and conversion
G	233	8.98	232	9.22	227	5.81	221	8.46	Carbohydrate transport and metabolism
E	244	9.41	247	9.82	246	9.22	242	9.27	Amino acid transport and metabolism
F	72	2.78	71	2.82	71	2.66	71	2.72	Nucleotide transport and metabolism
H	76	2.93	76	3.02	75	2.81	80	3.06	Coenzyme transport and metabolism
I	60	2.31	62	2.46	60	2.25	58	2.22	Lipid transport and metabolism
P	159	6.13	159	6.32	158	5.92	156	5.97	Inorganic ion transport and metabolism
Q	40	1.54	39	1.55	38	1.42	37	1.42	Secondary metabolites biosynthesis, transport and catabolism
R	320	12.34	310	12.33	318	11.92	307	11.76	General function prediction only
S	211	8.14	206	8.19	215	8.06	215	8.23	Function unknown
–	107	4.14	86	3.49	196	10.05	179	6.89	Not in COGs

The total is based on the total number of protein coding genes in the genome

A high degree of genomic sequence similarity among the four *B. thermosphacta* strains was observed by the calculation of Average Nucleotide Identity (ANI) using OrthoANIu, an orthologous ANI algorithm [13]. Strain to strain genomic comparisons showed orthoANI (Orthologous Average Nucleotide Identity) values varying from 98.94 to 99.11%, correlating thus with previous observations on other *B. thermosphacta* genome sequences [3, 4].

## Insights from the genome sequence

Comparative genomics of the pan genome was based on MicroScope gene/protein families (MICFAMs). This tool classifies proteins in homolog groups of proteins sharing at least 80% amino-acid identity and 80% alignment coverage [14]. The core genome includes MICFAMs associated with at least one gene from every compared genomes. The variable-genome includes MICFAM present in at least two compared genomes. Specific genome includes genes that are singletons and present in only one genome.

**Fig. 4:**
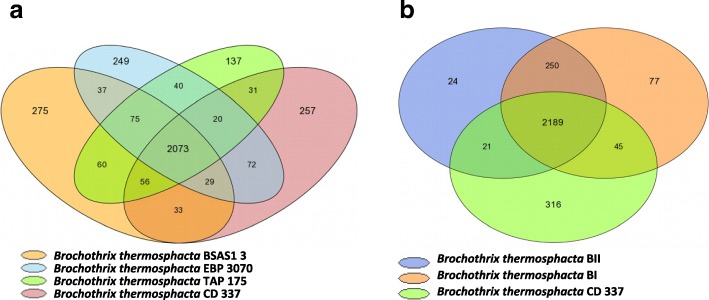
Core and pan genomes. Venn diagram analysis of (**a**) *Brochothrix thermosphacta* CD 337, *B. thermosphacta* TAP 175, *B. thermosphacta* BASA1 3, and *B. thermosphacta* EBP 3070; and (**b**) *B. thermosphacta* CD 337, *B. thermosphacta* BI, and *B. thermosphacta* BII. Values on diagram represent the numbers of MICFAM families for each organism intersections

The pan genome of strains CD337, TAP 175, BSAS1 3, and EBP 3070 comprised 10,373 genes. Among them, 8339 genes, corresponding to 2073 MICFAMs were shared by all strains and therefore represent the core genome. The variable genome contained 2034 genes grouped into 1371 MICFAMs. This analysis revealed that the four strains contain 10.19%, 5.46%, 10.66% and 9.59% strain-specific coding sequences, respectively. (Fig. 4a). The same analysis was performed on the 3 complete genomes, i.e. the newly sequenced genome CD337 and the two publicly available genomes of *B. thermosphacta* BI and BII, two strains isolated from ground chicken meat (Fig. 4b) [4]. From these 3 complete genomes, the pan genome comprised 7746 genes (2922 MICFAMs), with 6667 genes (2189 MICFAMs) constituting the core genome, and 1079 genes (733MICFAMs) the variable genome. Strain specific genome of *B. thermosphacta* BI, BII, and CD 337 comprised 3.21%, 0.94%, 12.06%, and genes, respectively. Strain-specific genes included some proteins involved in regulatory functions, cell surface composition, use of various carbon sources, or bacteriocin production. In particular we observed a four gene cluster, unique to *B. thermosphacta* CD 337 putatively encoding at least part of the machinery for the production of a type 2 lantibiotic. It encompasses a gene for putative lantibiotic modifying enzyme, lanthionine synthetase C-like, and lantibiotic leader peptide-processing serine protease, and a small 70 amino acid peptide similar to lichenicidin, a lantibiotic produced by *Bacillus licheniformis* [15]. However, a large proportion of these strain-specific genes encode proteins of unknown functions or are fragmented genes. The list of the specific genes of *B. thermosphacta* CD 337, BI, and BII is given in (Additional file 1: Table S1, Table S2, and Table S3, respectively).

Bacteriophage prediction results using PHAST (PHAge Search Tool) [16] showed that the four genomes contained at least one bacteriophage region. Similarities to phages previously described were provided based on the highest number of proteins most similar to those in the region. A schematic representation of the phage content of the four strains is presented in (Additional file 2: Figure S1). Both CD 337 and EBP 3070 harbored a complete phage (about 49 Kb) similar to LP-101 of *L. monocytogenes* [17] and an incomplete NF5 bacteriophage (18.8 Kb) previously described in *B. thermosphacta* [18]. CD 337 comprised a third region (18.8 Kb) similar to *L. monocytogenes* B054 phage [19]. TAP 175 genome comprised only one bacteriophage region consisting in a fraction of the *B. thermosphacta* NF5 bacteriophage. Strain BSAS1 3 harbored two complete bacteriophages similar to IME_SA4 and SANTOOR1 described in *Staphylococcus haemolyticus* and *Enterococcus faecalis*, respectively.

Since clustered regularly interspaced short palindromic repeats (CRISPR) and CRISPR-associated (Cas) proteins constitute an adaptive immune system against bacteriophages and other foreign genetic elements in bacteria and archaea [20], we investigate the occurrence of CRISPR-Cas systems in the four genomes of the present study. Interestingly, we found a diversity between strains regarding CRISPR/Cas System (Additional file 1: Table S4). Indeed, in the genome of the strain EBP 3070 we do not found CRISPR/Cas System, in the genome of the strain CD 337 we found only one putative CRISPR-associated endoribonuclease, while in the genome of the strain BSAS1 3 we found a CRISPR-Cas system composed by three CRISPR-associated endoribonucleases (Cas1, Cas2, and Cas9) and a Type II-A CRISPR-associated protein Csn2. Finally, in the genome of the strain TAP 175 we found a CRISPR-Cas system composed by at least 5 CRISPR-associated endoribonucleases (Cas1, Cas2, Cas4, Cas5d and Cas9) and a Type II-A CRISPR-associated protein Csn2. This diversity in four strains could participate to explain their adaptation and survival to various ecological niches, as CRISPR/Cas System provides bacterial immunity against lytic bacteriophages, which occur in food and food environments [21, 22].

*B. thermosphacta* CD 337 complete genome contained no plasmid. However, putative plasmids were found in the three draft genomes as shown in (Additional file 2: Figure S2). Indeed, these contained contigs harboring genes related to plasmid proteins (*ie: repB* involved in plasmid replication or *mob/pre* genes involved in recombination and conjugative mobilization). Such a 4557 bp long contig was found in both TAP 175 and BSAS1 3 with a high similarity degree between the two strains. This plasmid encoded also a protein annotated as a quaternary ammonium compound-resistance protein. The genome of EBP 3070 harbored two putative plasmids (8624 bp; 5011 bp). One of these may confer tetracycline resistance as it carried a gene encoding a multifunctional tetracycline antiporter which was 81% identical to the *tetB(L)* gene of *Bacillus subtilis* [23].

### Extended insights

Functions putatively involved in the niche adaptation and in the spoilage properties were searched in the genomes of the four strains. We found a large repertoire of substrate specific genes from the phosphoenolpyruvate dependent phosphotransferase system (PTS). The genomes of the four strains contained genes for glucose, maltose, fructose, mannose, trehalose, cellobiose, mannitol, beta-glucosides, and N-acetylglucosamine transport and phosphorylation. Genes encoding transporters for ribose, glycerol-3-phosphate, maltose, and *myo-*inositol were also present, attesting the large capacity of carbon sources used by *B. thermosphacta*.

No major difference for sugar utilization was noticed between the four strains. In addition predicted metabolic pathways of our strains were very similar to those previously described in other *B. thermosphacta* strains [3]. Briefly, all genes required for glycolysis and the pentose phosphate pathway were present in all strains. The citrate cycle was incomplete since only four of the eight enzymes were detected. The genes coding for alpha-ketoglutarate dehydrogenase (EC 1.2.4.2), succinate thiokinase (EC 6.2.1.4), succinate dehydrogenase (EC 1.3.5.1), and malate dehydrogenase (EC 1.1.1.37) were absent. Moreover, the gene coding the pyruvate carboxylase (EC 6.4.1.1) was present while that of the fumarate reductase (EC 1.3.5.4) was absent.

Genes involved in the production of molecules associated to meat or seafood spoilage, as lactate, ethanol, acetate, acetoin, diacetyl, and 2.3-butanediol were identified. Although we had previously observed differences between strains in their ability to produce acetoin, the *butA* gene encoding diacetyl reductase [(S)-acetoin forming], as well as its 300 bp upstream region was 100% identical in all strains. Conversely, the *bdhA* gene encoding (R,R)-2,3 butanediol dehydrogenase able to convert (R,R)- 2,3-butanediol or diacetyl to acetoin presented 100% identity between BSAS1 3 and CD 337 but, amino-acid at position 201 (alanine) was replaced by glutamic acid in both EBP 3070 and TAP 175 enzymes, thus introducing a negative charge. Interestingly replacing aspartic acid by alanine in glycerol dehydrogenase of *Escherichia coli* improved its activity toward 1,3-butanediol [24]. As EBP 3070 and TAP 175 had a similar pattern for acetoin and diacetyl production (they were among the highest producers of acetoin and diacetyl in shrimp juice, and among the lowest in beef) we can hypothesize that the alanine/glutamate replacement may be involved in the different acetoin production levels we previously observed (Fig. S3).

Moreover, the upstream region of the *bdhA* gene of EBP 3070 and TAP 175 were identical and showed differences with that of BSAS1 3 at positions − 141, − 137, − 110, − 67, and − 43 upstream from the start codon suggesting transcriptional regulation of *bdhA* might be different. However, CD 337 presented the same differences as EBP 3070 and TAP 175 plus an additional one at position − 46.

**Fig. 5:**
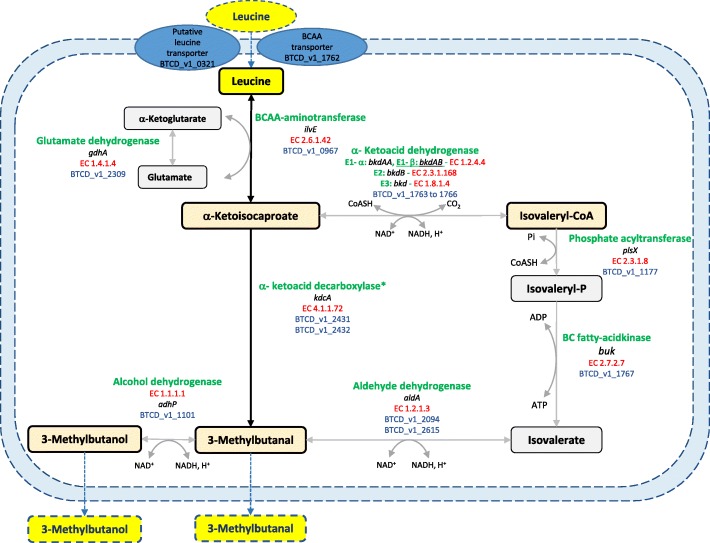
Probable metabolic pathway for the biosynthesis of 3-methylbutanal from l-leucine degradation by *B. thermosphacta.* All the genes encoding the necessary enzymes for the direct (dark line) and the indirect (gray line) pathways were present. Alcohol dehydrogenase was also present. BCAA refrers to branched-chain amino acids. *The *kdcA* gene is fragmented in *B. thermosphacta* CD 337. The underlined gene *bkdAB* is fragmented in BSAS1 3

In addition, genes involved in the production of isovaleric, isobutyric, and 2-methylbutyric branched-chain fatty acids were found in the genome of the four strains. These compounds, associated with off-odors, were suggested to be produced from the degradation of branched-chain amino acids leucine, valine, and isoleucine, respectively [25]. The catabolism of leucine can also lead further to the synthesis of 3-methylbutanal and 3-methylbutanol. 3-methybutanal, a branched-chain aldehyde has been described as associated to the production of desirable aroma in many cheeses [26], while it is responsible for off-odors in meat and seafood products [27, 28]. The pathway of 3-methylbutanal production from leucine catabolism has been described in lactic acid bacteria. The first step of this pathway is the transamination of leucine to α-ketoisocaproate which is the central metabolite in leucine degradation [29]. Then the formation of 3-methylbutanal may occur in two possible metabolic pathways: directly via the non-oxidative decarboxylation by an α-ketoacid decarboxylase or indirectly through an oxidative decarboxylation by α-ketoacid dehydrogenase [26]. Investigation of *B. thermosphacta* genomes showed that all the genes encoding enzymes required for isovalerate, 3-methylbutanal and 3-methylbutanol production from leucine were present (Fig. 5). Interestingly, the α-ketoacid decarboxylase from BSAS1 3, TAP 175, and EBP 3070 was 100% identical (except glutamine 311 replaced by a histidine in EPB 3070) but was mutated in CD 337 resulting in a fragmented gene and 2 mutations on amino acids 200 and 205. We also noticed that the gene encoding the E1 component, β subunit of the α-keto acid dehydrogenase was 100% identical in CD 337, EPB 3070, and TAP 175 but fragmented in BSAS1 3. This suggests that production of isovalerate, 3-methylbutanol, and 3-methylbutanal may differ between strains and use different pathways, possibly impacting the spoilage potential of the strains.

Since *B. thermosphacta* has been associated to the production of biogenic amines [30] the presence of amino acid decarboxylases was searched in the genomes. Genes encoding histidine decarboxylase and tyrosine decarboxylase responsible for the production of histamine and tyramine, respectively, were not found. Nevertheless, all the genomes harbored the genes encoding the ABC transporter complex PotABCD involved in the import of spermidine and putrescine, two polyamines present in meat and fish.

A putative cell-wall associated adhesin was found in CD 337 genome, which was absent or truncated in other strains. Adhesins may promote substrate adhesion and survival in the environment, and the presence of adhesin in only some strains may contribute to their different niche adaptation. Nine proteins annotated as containing an LPxTG-like motif were detected in CD 337. In addition, among the genes encoding exported proteins of unknown functions two CDS (BTCD_v1_656 and BTCD_v1_1960) also harbored an LPxTG motif and a signal peptide, as well as a protein annotated as a putative fimbrial isopeptide formation D2 domain-containing protein (BTCD_v1_1958). Such proteins are surface proteins covalently linked to the bacterial cell wall by a sortase. All were well conserved in the four strains, except three (BTCD_v1_1958 to 1960) which were unique to CD 337 and located in a genomic island of 24,132 bp. This island also encompassed genes encoding putative recombinases and transposases suggesting its acquisition through horizontal gene transfer. Putative sortase genes were also present, showing this island may indeed encode functions for specifically linking the three LPxTG proteins to the cell surface. It also hosted genes encoding proteins involved in polysaccharide metabolism as a putative polysaccharide deacetylase, a putative polysialyltransferase, and proteins resembling the NeuBCDA enzymes involved in the amino sugar N-acetyl neuraminic acid (sialic acid) metabolism. Futhermore, a putative O-acetyltransferase EpsM (BTCD_v1_0680) most probably involved in biofilm formation was also found in CD 337 genome, conserved in *B. thermosphacta* BI and BII genomes, but absent in the three other strain genomes of the present study (EPB 3070, TAP 175 and BSAS1 3). It has been shown in *B. subtilis* a similar O-acetyltransferase (EpsM), which is a member of the eps operon, involved in the production of the exopolysaccharide (EPS) component of the extracellular matrix during biofilm formation [31].

All these proteins may have an important role in the survival and the persistence of bacteria in the food-processing environment. Such differences in the gene repertoire between the four strains might correlate to the different substrates our strains were isolated from.

## Conclusions

The four strains we selected for comparative genomics were chosen as diverse as possible (different ecological origin, different ability to produce some spoilage molecules, and belonging to different PFGE and Rep-PCR clusters). However, a high genome content similarity was observed as previously reported by other authors on different meat product issued strains. The major differences we observed in the gene content were represented by phages or plasmids, restriction/modification systems, cell surface functions, or use of various carbon sources. These could participate to their fitness or adaptation to various niches, in particular the functions involved in carbon sources utilization or those associated to cell surface or adhesion that may help to colonize specific environment. Most of the strain specific genome encompasses proteins of unknown function.

The simple comparison of the variable genome could not explain the differences we observed in the ability to produce acetoin and diacetyl. Nevertheless, we showed that mutations (fragmentation or point mutation) in genes encoding enzymes involved in the production of VOCs and differences in the DNA sequences located upstream from start codons, thus potentially in the promoter region of these genes may lead to different efficacies to produce such VOCs and therefore to spoil meat or seafood products.

Therefore, the diversity of spoilage potential of *B. thermosphacta* on various foods reported in the literature may result from i) a strain dependent specificity to adapt to different ecological niches, characterized by strain specific genome content; ii) a strain dependent capacity to produce malodorous molecules driven by the presence/absence/mutations of enzymes involved in the catabolism of branched chain amino acids and pyruvate; and iii) a strain dependent capacity to express the corresponding genes.

## Additional files

Additional file 1: List of the specific genes of *B. thermosphacta* CD 337 (**Table S1)**, BI (**Table S2)**, and BII (**Table S3)**. **Table S4.** List of CRISPR genes found in CD 337, BSAS1 3, and TAP 175. (XLSX 41 kb)

Additional file 2: **Figure S1.** Schematic representation of phage content of the four *B. thermosphacta* strains. The phage identification was given by PHAST program [16]. It refers to the phage with the highest number of proteins most similar to those in the region. The phages were represented by boxes surrounded by solid line (intact phages) or dashed line (incomplete phages). The phage size and the number of CDS were also given. **Figure S2.** Schematic representation of putative plasmids content of three *B. thermosphacta* strains. The plasmids size and the CDS content were given. **Figure S3.** Metabolic pathway for the production of acetoin and diacetyl from pyruvate degradation. All genes encoding the necessary enzymes were found. (PPTX 47 kb)

## Abbreviations

CFU: Colony forming unit
KEGG: Kyoto Encyclopedia of Genes and Genomes
MALDI-TOF: Matrix assisted laser desorption ionization - time of flight
MICFAM: MicroScope Family
PFGE: Pulsed-field gel electrophoresis
PkGDB: Prokaryotic Genome DataBase:
Rep-PCR: Repetitive element palindromic based-polymerase chain reaction
STAA: Streptomycin-thallous acetate-actidione
VOCs: Volatile organic compounds
