# Supplementary material for: One complete and three draft genome sequences of four Brochothrix thermosphacta strains, CD 337, TAP 175, BSAS1 3 and EBP 3070
Source: Stand Genomic Sci. 2018 Oct 10;13:22. doi: 10.1186/s40793-018-0333-z (PMC6180393; doi:10.1186/s40793-018-0333-z)
Supplement: Supplementary file 2 — Figure S1. Schematic representation of phage content of the four B. thermosphacta strains. The phage identification was given by PHAST program [16]. It refers to the phage with the highest number of proteins most similar to those in the region. The phages were represented by boxes surrounded by solid line (intact phages) or dashed line (incomplete phages). The phage size and the number of CDS were also given. Figure S2. Schematic representation of putative plasmids content of three B. thermosphacta strains. The plasmids size and the CDS content were given. Figure S3. Metabolic pathway for the production of acetoin and diacetyl from pyruvate degradation. All genes encoding the necessary enzymes were found. (PPTX 47 kb) [file 40793_2018_333_MOESM2_ESM.pptx]

## Slide 1
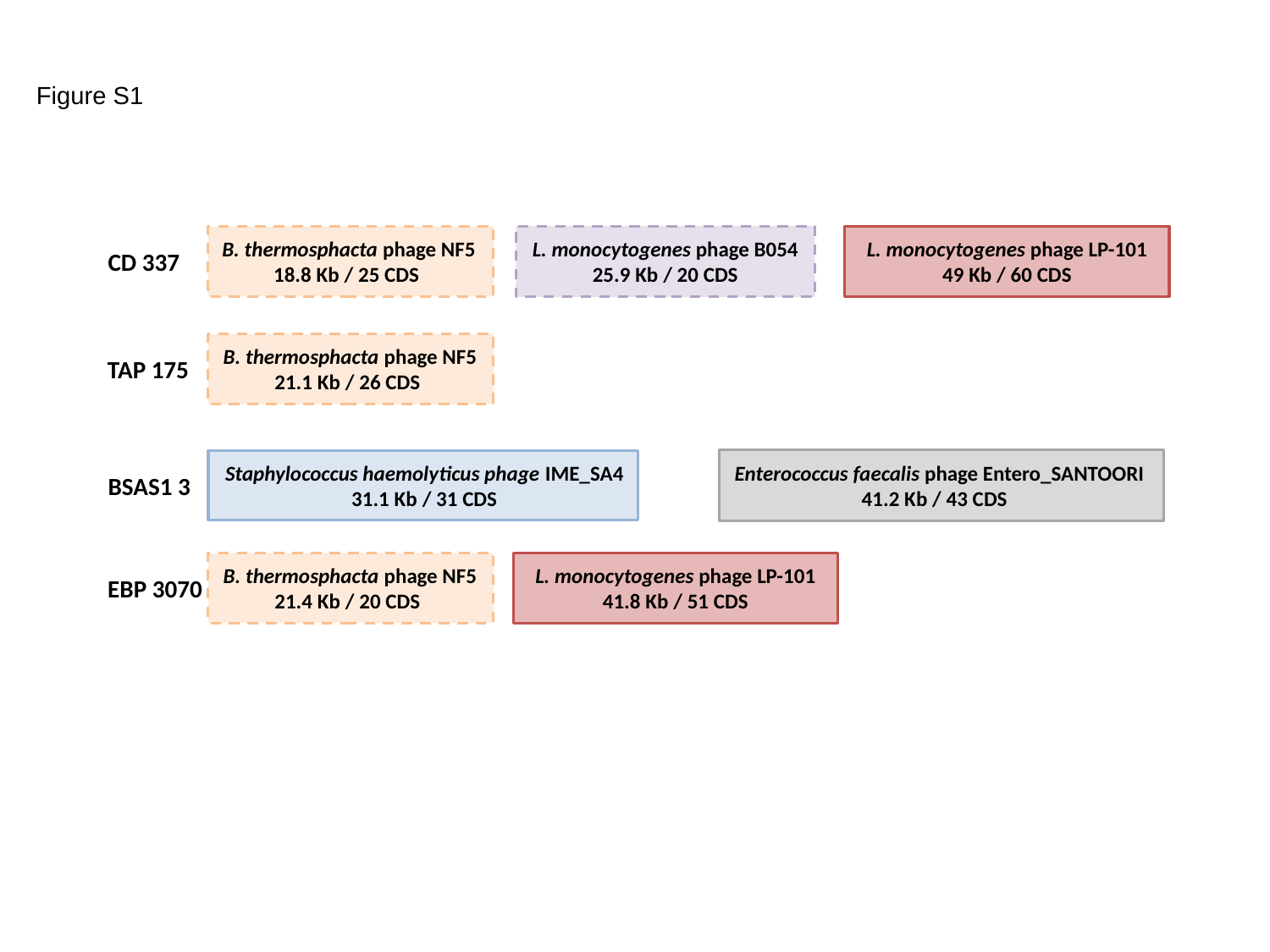

Figure S1
B. thermosphacta phage NF5
18.8 Kb / 25 CDS
L. monocytogenes phage B054
25.9 Kb / 20 CDS
L. monocytogenes phage LP-101
49 Kb / 60 CDS
CD 337
B. thermosphacta phage NF5
21.1 Kb / 26 CDS
TAP 175
Staphylococcus haemolyticus phage IME_SA4 31.1 Kb / 31 CDS
Enterococcus faecalis phage Entero_SANTOORI 41.2 Kb / 43 CDS
BSAS1 3
B. thermosphacta phage NF5
21.4 Kb / 20 CDS
L. monocytogenes phage LP-101
41.8 Kb / 51 CDS
EBP 3070

## Slide 2
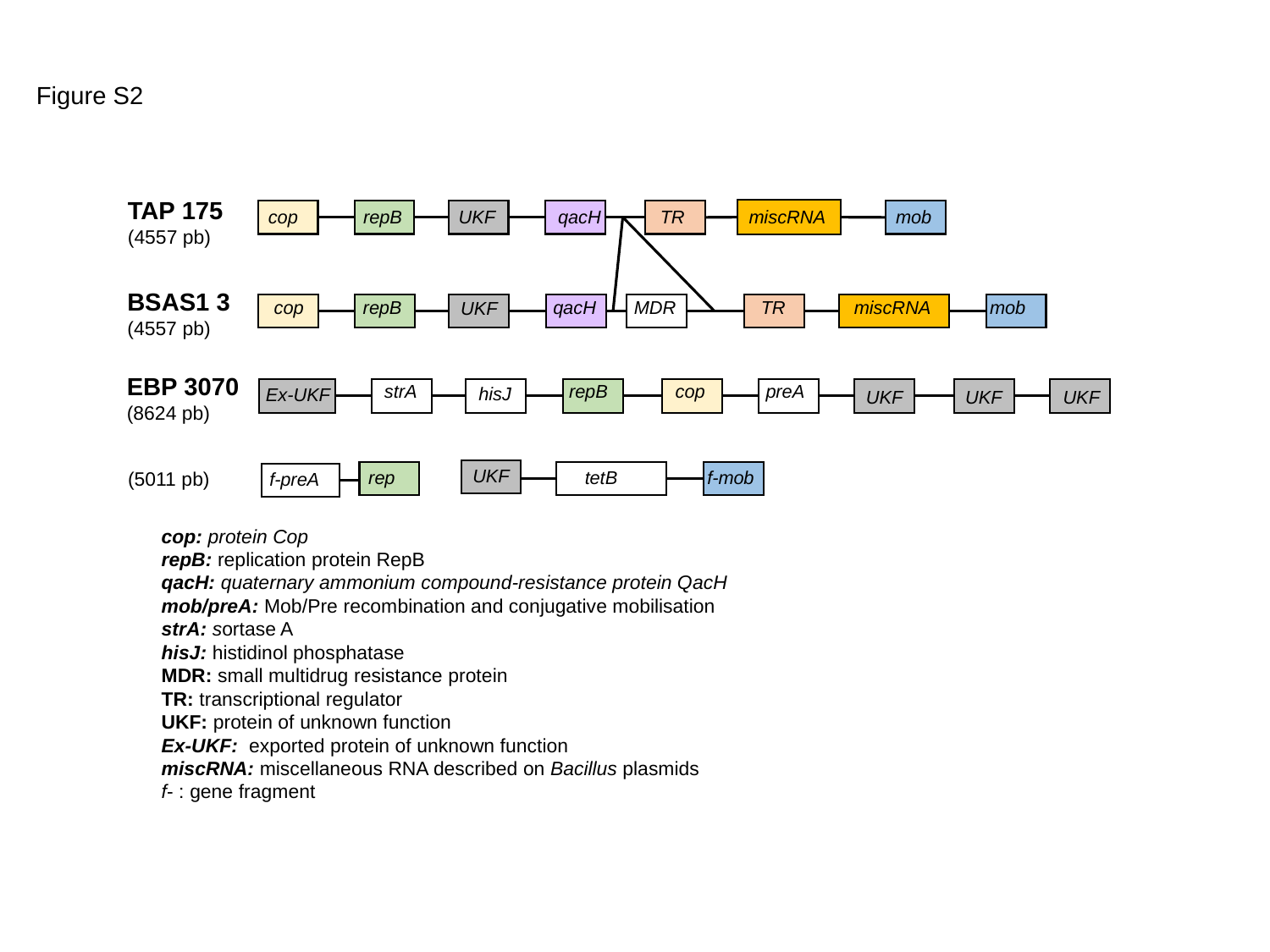

Figure S2
TAP 175
(4557 pb)
cop
repB
UKF
qacH
TR
miscRNA
mob
BSAS1 3
(4557 pb)
cop
repB
qacH
MDR
TR
miscRNA
mob
UKF
EBP 3070
(8624 pb)
strA
repB
cop
preA
hisJ
Ex-UKF
UKF
UKF
UKF
UKF
tetB
f-mob
rep
(5011 pb)
f-preA
cop: protein Cop
repB: replication protein RepB
qacH: quaternary ammonium compound-resistance protein QacH
mob/preA: Mob/Pre recombination and conjugative mobilisation
strA: sortase A
hisJ: histidinol phosphatase
MDR: small multidrug resistance protein
TR: transcriptional regulator
UKF: protein of unknown function
Ex-UKF: exported protein of unknown function
miscRNA: miscellaneous RNA described on Bacillus plasmids
f- : gene fragment

## Slide 3
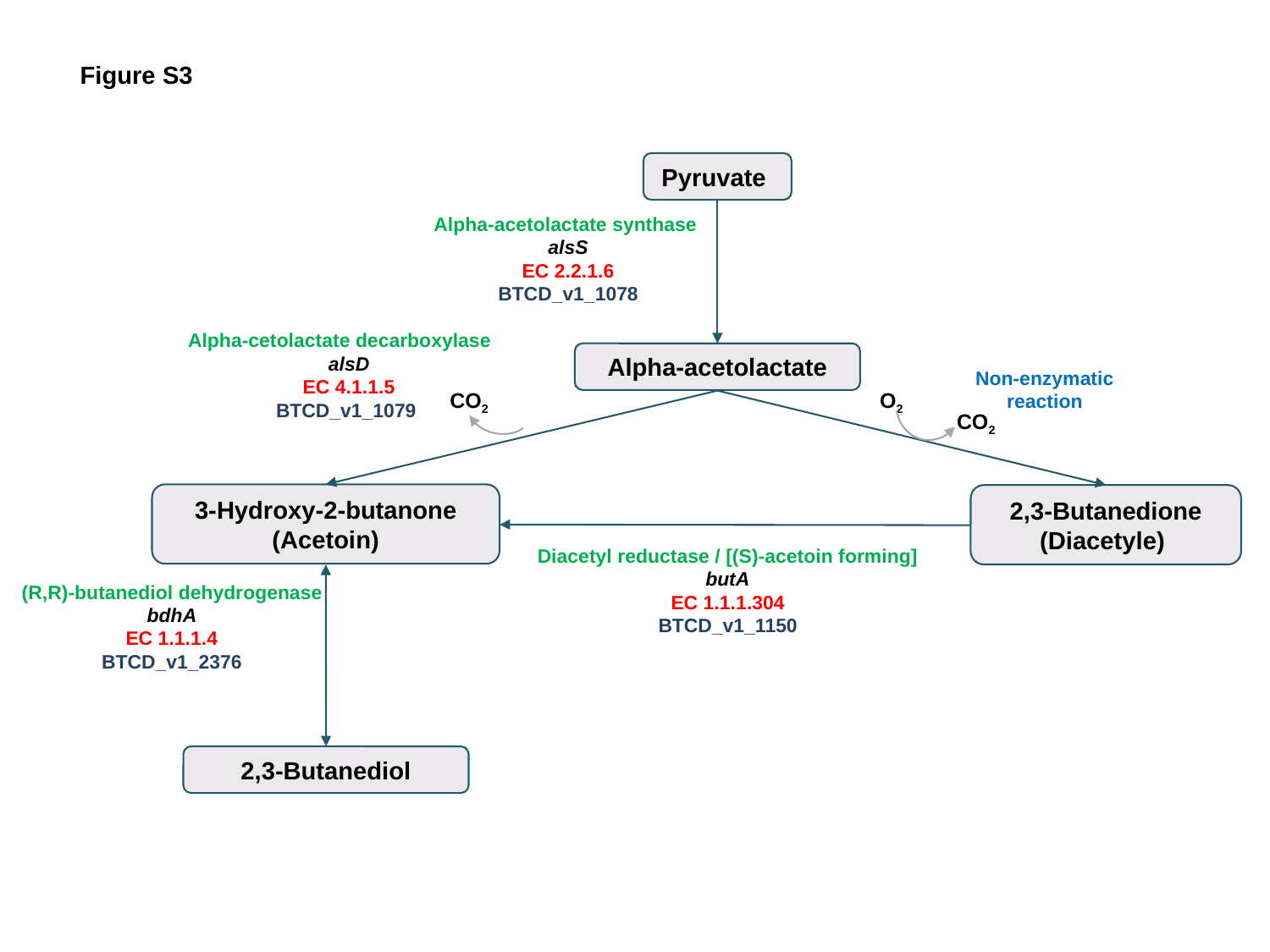

Figure S3
Pyruvate
Alpha-acetolactate synthase
alsS
EC 2.2.1.6
BTCD_v1_1078
Alpha-cetolactate decarboxylase
 alsD
 EC 4.1.1.5
BTCD_v1_1079
Alpha-acetolactate
O2
CO2
CO2
3-Hydroxy-2-butanone (Acetoin)
2,3-Butanedione
(Diacetyle)
Diacetyl reductase / [(S)-acetoin forming]
butA
EC 1.1.1.304
BTCD_v1_1150
(R,R)-butanediol dehydrogenase
bdhA
EC 1.1.1.4
BTCD_v1_2376
2,3-Butanediol
Non-enzymatic reaction
